# Protective Effect of Increased Zinc Supply against Oxidative Damage of Sublingual Gland in Chronic Exposure to Cadmium: Experimental Study on Rats

**DOI:** 10.1155/2018/3732842

**Published:** 2018-07-04

**Authors:** Paula Kostecka-Sochoń, Barbara M. Onopiuk, Ewa Dąbrowska

**Affiliations:** ^1^Department of Restorative Dentistry, Medical University of Białystok, Białystok, Poland; ^2^Medical University of Białystok and Private Dental Practice in Białystok, Białystok, Poland; ^3^Department of Social and Preventive Dentistry, Medical University of Białystok, Białystok, Poland

## Abstract

Cadmium is one of the main chemical pollutants found in the daily environment of developed countries. Cigarettes are a significant source of that metal, which makes it important in terms of oral cavity health. The aim of this study was to determine if increased supply of zinc in chronic exposure to cadmium might protect the sublingual gland structure against oxidative damage. The experiment took 12 months and was conducted on 72 adult male rats. They were randomized into 9 groups. Eight groups received cadmium in drinking water (as CdCl_2_) at 5 or 50 mg Cd/dm^3^ and/or zinc (as ZnCl_2_) at 30 or 60 mg Zn/dm^3^. The control group received regular water. In the sublingual gland of all animal groups, levels of oxidative parameters were measured. The oxidative stress index was calculated as a TOS/TAS ratio. Cadmium exposure at 5 mg and 50 mg Cd/dm^3^ induced oxidative stress in the sublingual glands of the rats. Cadmium reduced the TAS and GSH levels and increased LPO, H_2_O_2_, TOS, and OSI. In cadmium exposure conditions, increasing the supply of zinc by 79% or 151%, as compared to the standard dietary intake of this microelement, completely prevented the reduction of TAS and GSH levels and accumulation of LPO, H_2_O_2_, and TOS in the examined gland at both exposure levels to that metal. The outcome data confirm the protective effect of increased zinc intake on the sublingual gland tissue in chronic cadmium exposure.

## 1. Introduction

Cadmium is one of the main chemical pollutants found in the daily environment of developed countries [1–4]. Cigarettes are a significant source of this metal, which makes it important in terms of oral cavity health [4–6].

Cadmium is accumulated in various tissues and organs and may have serious consequences for the general population health. Chronic exposure to cadmium may damage the kidneys, bones, liver, lungs, and other organs, including sublingual glands [7–12]. An important role in the cadmium activity mechanism is attributed to its strong prooxidative properties [9, 13–15]. Cadmium has the ability to form reactive oxygen species (ROS) through direct impact, that is, by disrupting the electron transport chain, or indirectly by weakening the enzymatic and nonenzymatic antioxidative barrier [8, 9, 13, 15]. Disruptions of the cellular oxidation-reduction balance may cause damage to tissues and organs and impair their functions [8, 12, 16, 17].

The available literature of the subject indicates that many toxic effects of cadmium can be prevented or at least reduced by increasing the supply of zinc [8, 18–21], as zinc exhibits antagonistic activity towards that toxic metal. It has been shown that this bioelement is able to provide efficient protection against damage to the organs in which cadmium accumulates the most, that is, the liver, kidneys, and bones [10, 19, 20, 22]. It is believed that the protective role of zinc against cadmium toxicity arises from the former's antioxidant properties [21, 23–26].

Thus far, the impact of zinc on the sublingual gland of an organism exposed to cadmium has not been studied. In consideration thereof, this study involved experimental analysis to determine if the increased supply of zinc in chronic exposure to cadmium might protect the sublingual gland structure against oxidative damage. For this purpose, in the sublingual gland of rats which received cadmium and/or zinc and of control animals, the oxidative stress markers were assayed.

## 2. Materials and Methods

The experiment took 12 months and was conducted on 72 adult male Wistar rats with an initial body weight of 220 g. Throughout the experiment period, the animals were kept in standard conditions (air temperature 18–21°C, relative humidity 50 ± 10%, and 12-hour circadian rhythm) and were provided with unlimited access to balanced granulated LSM fodder (Motycz near Lublin) and drinking water.

The research protocol was approved by the Local Ethics Committee for Animal Experiments in Białystok (Poland) and performed in accordance with the ethical principles and institutional guidelines and International Guide for the Use of Animals in Biomedical Research.

The rats were randomized into 9 groups, and in each group was 8 animals. In research, 2 groups received Zn alone, 2 groups were treated with Cd alone, and 4 groups received Zn supplementation during exposure to Cd. Zn and Cd were administered in drinking water at the concentrations of 30 or 60 mg Zn/L (as ZnCl_2_; Merck) and 5 or 50 mg Cd/L (as CdCl_2_·2 1/2H_2_O; POCH; Gliwice, Poland) alone (30 mg Zn/L, 60 mg Zn/L, 5 mg Cd/L, and 50 mg Cd/L groups) and in combination (5 mg Cd/L + 30 mg Zn/L, 5 mg Cd/L + 60 mg Zn/L, 50 mg Cd/L + 30 mg Zn/L, and 50 mg Cd/L + 60 mg Zn/L groups) for up to 12 months. The control group received drinking water without cadmium or zinc. During the experiment, the daily intake of fluids and body weight gain were controlled. Both the fluid intake and body weight gains were similar across all rat groups. Rat exposure to cadmium at 5 mg/dm^3^ is an equivalent of environmental exposure of humans to that metal, particularly smokers; at 50 mg of Cd/dm^3^, it is equivalent to occupational exposure and exposure arising from high pollution and heavy smoking. Administering zinc at 30 mg or 60 mg/dm^3^ to the animals increased the daily intake of this bioelement by 79% and 151%, respectively, in comparison with standard dietary intake. This dose was chosen based on findings of other authors and observations of the Department of Toxicology of the Medical University of Białystok [10, 19].

After ending the exposure, the animals were put under barbiturate anesthesia (Vetbutal 30 mg/kg of body weight i.p.) and various types of biological material were collected for analysis, including sublingual glands which were flushed in PBS, drained on blotting paper, and secured for further analysis by deep freezing at −80°C. After thawing, the dissected glands were weighted and 20% homogenates were prepared using a glass tissue homogenizer (Schuett Homogen, Göttingen, Germany) in a cold 50 mM potassium phosphate buffer with pH = 7.4. In order to prevent automatic oxidation of the analyzed material, 0.5 M BHT acetonitrile was added to the samples (10 *μ*L per 1 cm^3^ of homogenate). The homogenates were centrifuged (MPW-350R, Medical Instruments laboratory centrifuge; Warsaw, Poland) 700 ×g for 20 minutes at 4°C. After centrifugation, the supernatant was immediately isolated and the oxidative stress markers and protein levels were assayed [27].

The total antioxidative capacity (TAS) and the total oxidative status (TOS) of the homogenates were determined using ImAnOx (TAS) ELISA kit and PerOx (TOS) ELISA kit by Immundiagnostik AG (Germany). The TAS values assayed in the control samples provided with the kit were 191.88 ± 10.7 and 264.33 ± 15.6 *μ*mol/L (average ± SEM; *n* = 2) and fell within the range of values provided by the manufacturer (170–230 *μ*mol/L and 258–350 *μ*mol/L). The precision of the method expressed as a coefficient of variation (CV) was <6%. The TOS values assayed in the control samples provided with the kit were 156.36 ± 4.27 and 424.85 ± 10.16 *μ*mol/L (average ± SEM; *n* = 2) and fell within the range of values provided by the manufacturer (170–230 *μ*mol/L and 305–509 *μ*mol/L). The precision of the method expressed as CV was <3%.

The glutathione (GSH) levels were assayed using the Glutathione Assay Kit, Cayman Chemical (USA). The precision of the method expressed as CV was <1.5%.

The lipid peroxidation (LPO) levels (Bioxytech® LPO-586™) and hydrogen peroxide (H_2_O_2_) (Bioxytech® H_2_O_2_-560™) were assayed using kits supplied by OxisResearch (USA). The precision of the method expressed as CV was <4.5%.

All assays performed using commercial kits were performed as per the manufacturers' instructions, and the measured parameters were adjusted for protein concentration.

The obtained results were analyzed statistically using Statistica 10 software (StatSoft; Tulsa, USA). In order to assess the statistical significance of differences between the study groups, a one-way analysis of variance (ANOVA) was performed using Duncan's post hoc test. The independent and interactive impact of cadmium and zinc on the stress index levels was assessed using a two-way analysis of variance (ANOVA/MANOVA). Also, Spearman's rank correlation test was performed for the assessed parameters in the tissue of the studied gland. The differences between groups and correlations between variables were considered statistically significant at *p* < 0.05.

## 3. Results

### 3.1. The Impact of Zinc and/or Cadmium on Glutathione (GSH) Levels in the Rat Sublingual Gland

The GSH levels in the sublingual gland of the rats are provided in Table 1. The animals' exposure to 5 mg or 50 mg Cd/dm^3^ resulted in the reduction of GSH levels by 24% (*p* < 0.001) and 29% (*p* < 0.01), respectively, as compared to the control group. In the rat group which, throughout the period of exposure to cadmium at 5 mg/dm^3^, received 60 mg Zn/dm^3^, the GSH levels were 24% (*p* < 0.01) higher as compared to the GSH levels in the control group, whereas for zinc received in 30 mg/dm^3^ doses, the GSH levels remained unchanged. In the animal groups which, throughout the period of exposure to cadmium at 5 mg/dm^3^, received 30 mg or 60 mg Zn/dm^3^, the GSH levels were 39% (*p* < 0.01) and 63% (*p* < 0.001) higher, respectively, as compared to the GSH levels in the animals exposed solely to cadmium. Also, in the rat groups which, throughout the period of exposure to cadmium at 50 mg/dm^3^, received 30 mg or 60 mg Zn/dm^3^, the GSH levels were 28% (*p* < 0.05) and 30% (*p* < 0.05) higher, respectively, as compared to the GSH levels in the animals exposed to cadmium.

### 3.2. The Impact of Zinc and/or Cadmium on Oxidative Stress Index (Lipid Peroxidation and Hydrogen Peroxide) in the Rat Sublingual Gland

Exposure of rats to 5 mg or 50 mg Cd/dm^3^ resulted in the increase of LPO levels 2.2-folds (*p* < 0.01) and 3-folds (*p* < 0.001), respectively, as compared to the control group; the increase was larger at a higher exposure level. In the rat groups which, throughout the period of exposure to cadmium at 5 mg/dm^3^, received 30 mg or 60 mg Zn/dm^3^, the LPO levels were 2.3- and 2.5-folds lower (*p* < 0.01), respectively, as compared to the LPO levels in the animals exposed to cadmium and did not differ from the values observed in the control group. Also, in the rat groups which, throughout the period of exposure to cadmium at 50 mg/dm^3^, received 30 mg or 60 mg Zn/dm^3^, the LPO levels were 38% (*p* < 0.01) and 56% (*p* < 0.001) lower, respectively, as compared to the LPO levels in the animals exposed to cadmium. In the animal group which, throughout the period of exposure to cadmium at 50 mg/dm^3^, received 30 mg Zn/dm^3^, the LPO levels were higher than the LPO levels in the control group but did not differ at a higher level of supplementation of that bioelement (Table 1).

Supplementation of zinc at 30 mg/dm^3^ had no impact on the H_2_O_2_ levels in the analyzed gland, but a higher concentration of that bioelement would reduce it by 26%. Exposure of rats to 5 mg or 50 mg Cd/dm^3^ resulted in the increase of H_2_O_2_ levels by 26% (*p* < 0.05) and 96% (*p* < 0.001), respectively, as compared to the control group; the increase was larger at a higher exposure level. In the rat groups which, throughout the period of exposure to cadmium at 5 mg/dm^3^, received 30 mg or 60 mg Zn/dm^3^, the H_2_O_2_ levels were 35% and 33% lower (*p* < 0.001), respectively, as compared to the H_2_O_2_ levels in the animals exposed to cadmium and did not differ from the values observed in the control group. Also, in the rat groups which, throughout the period of exposure to cadmium at 50 mg/dm^3^, received 30 mg or 60 mg Zn/dm^3^, the H_2_O_2_ levels were 48% and 58% lower (*p* < 0.001), respectively, as compared to the H_2_O_2_ levels in the animals exposed to cadmium and did not differ from the values observed in the control group (Table 1).

### 3.3. The Impact of Zinc and/or Cadmium on Total Oxidant State and Total Antioxidant Status and the Stress Index in the Rat Sublingual Gland

The TOS levels in the sublingual gland of rats are provided in Table 2. Exposure to cadmium at 5 mg and 50 mg/dm^3^ resulted in significant increase of TOS levels in the sublingual gland. The TOS levels in both groups were 1.7- and 2-folds higher (*p* < 0.001) than in the control group. Furthermore, the TOS levels in rats exposed to 50 mg Cd/dm^3^ was 1.2-folds higher (*p* < 0.05) than in those exposed to lower cadmium levels. In the animal groups which, throughout the period of exposure to cadmium at 5 mg/dm^3^, received 30 mg or 60 mg Zn/dm^3^, the TOS levels were 46% and 42% lower (*p* < 0.001), respectively, as compared to the TOS levels in the animals exposed solely to cadmium. Also, in the rat groups which, throughout the period of exposure to cadmium at 50 mg/dm^3^, received 30 mg or 60 mg Zn/dm^3^, the TOS levels were 52% and 40% lower (*p* < 0.001), respectively, as compared to the TOS levels in the animals exposed to cadmium and did not differ from the values observed in the control group.

The TAS levels in the sublingual gland of rats which, throughout the experiment period, received only zinc at 30 mg or 60 mg/dm^3^ were 2.3- and 1.8-folds higher (*p* < 0.001), respectively, than the TAS levels in control animals. Exposure to 5 mg and 50 mg Cd/dm^3^ resulted in significant decrease of TAS levels by 49% and 44% (*p* < 0.05), respectively, as compared to the control group. In the rat groups which, throughout the period of exposure to cadmium at 5 mg/dm^3^, received 30 mg or 60 mg Zn/dm^3^, the TAS levels were 2.3- and 3.5-folds higher (*p* < 0.001), respectively, than The TAS levels in the control group and 4.5- and 6.8-folds higher (*p* < 0.001) as compared to The TAS levels in the animals not receiving zinc during exposure to cadmium. Also, in the rat groups which, throughout the period of exposure to cadmium at 50 mg/dm^3^, received 30 mg or 60 mg Zn/dm^3^, the TAS levels were 1.6- and 1.5-folds higher (*p* < 0.05), respectively, than the TAS levels in the control group and 2.8- and 2.6-folds higher (*p* < 0.001) as compared to the TAS levels in the animals not receiving zinc during exposure to cadmium (Table 2).

In the animals which, throughout the experiment, received only zinc at 30 mg and 60 mg/dm^3^, the TOS/TAS ratio was 3.5-folds (*p* < 0.01) and 2.4-folds (*p* < 0.05) lower, respectively, than the TOS/TAS ratio in the control groups. Exposure to 5 mg and 50 mg Cd/dm^3^ resulted in a significant increase of the TOS/TAS ratio in the sublingual gland. The TOS/TAS ratio in both groups was 2.7- and 3.1-folds higher (*p* < 0.001) than that in the control group. Furthermore, the TOS/TAS ratio in both groups was 9.4- and 6.3-folds, which was significantly higher as compared to that in animals which received only zinc with drinking water at 30 mg and 60 mg/dm^3^ (*p* < 0.001), respectively, when exposed to 5 mg Cd/dm^3^, and 11.1- and 7.4-folds higher (*p* < 0.001), respectively, when exposed to 50 mg Cd/dm^3^. The TOS/TAS ratio was significantly higher in the group of animals receiving cadmium at 50 mg/dm^3^ than in the group exposed to 5 mg Cd/dm^3^ by 15% (*p* < 0.05). In the rat groups which, throughout the period of exposure to cadmium at 5 mg/dm^3^, received 30 mg or 60 mg Zn/dm^3^, the TOS/TAS ratio was 67% and 77% lower (*p* < 0.01), respectively, than the TOS/TAS ratio in the control group and 8.2- and 11.6-folds lower (*p* < 0.001), respectively, as compared to the TOS/TAS ratio in the animals receiving only cadmium. The TOS/TAS ratio in the rat groups which, throughout the period of exposure to 50 mg Cd/dm^3^, received 30 mg or 60 mg Zn/dm^3^ was 5.9- and 4.5-folds lower (*p* < 0.001), respectively, than in the group of animals receiving only cadmium (Table 2).

### 3.4. Independent and Interactive Impact of Cadmium and Zinc on the Levels of Selected Oxidative Stress Indices in the Rat Sublingual Gland (Table 3)

The independent and interactive impact of cadmium and zinc on the levels of selected stress indices was assessed using a two-way analysis of variance (ANOVA/MANOVA).

### 3.5. Analysis of Spearman's Rank Correlation between the Assessed Parameters in the Sublingual Gland Tissue (Table 4)

## 4. Discussion

Cadmium is a toxic metal commonly found in the daily environment. Due to the increasing number of reports of the harmful impact of low exposure to those toxic elements published worldwide, the researchers are focused on finding methods to reduce dietary cadmium intake and mitigate its impact on the organism [2, 8, 10]. Particular attention is being paid to using certain nutrients for this purpose, including zinc. Studies on animals revealed that zinc reduces cadmium absorption from the gastrointestinal tract and its accumulation in the liver and the kidneys, and it prevents some effects of cadmium, in particular its hepato- and nephrotoxicity and toxicity to the bones [10, 19].

Similar to other heavy metals, cadmium has the ability to accumulate in living organisms. The largest accumulation of cadmium occurs in organs rich in metallothionein (MT), that is, the liver and kidneys [10, 28, 29]. However, in chronic exposure to cadmium, the concentration levels of this metal in various tissues and body fluids increased, including those in which the accumulation is much lower than in shielding organs, for example, in the salivary gland tissue [30, 31].

The oral cavity, serving as the initial section of the gastrointestinal tract, is an integral element of the organism. Its liquid environment is the saliva which hosts a number of biochemical reactions necessary to maintain the oral cavity healthy. Exposure to cadmium also affects its condition. Consequences of chronic exposure to that toxic element were also observed both in hard tissue, such as teeth [5, 22, 32], and in soft tissue of the oral cavity and in the saliva [7, 30, 31, 33, 34].

Fischer et al. [5] assessed the presence of bioelements and toxic metals in milk teeth of children exposed to tobacco smoke from cigarettes smoked by their parents. In the milk teeth of children who had not been exposed to tobacco smoke, a higher content of elements with a confirmed physiological role for the organism (Fe, Zn, K, Na, and Ca) was found, as compared to the content of those metals in the milk teeth of children subjected to passive exposure. On the other hand, the content of toxic metals (Pb and Cd) was higher (*p* = 0.05) in the teeth's tissue of children exposed to passive smoking. The authors suggest that exposure to tobacco smoke causes the toxic metals in the smoke to be integrated with the structure of mineralized tissues, including teeth [5].

Kakei et al. [22] concluded that cadmium disrupted the formation of tooth enamel in rats by inhibiting the crystallization buds in the form of zinc phosphate. The authors suggested that Cd^2+^ ions, by replacing Zn^2+^ ions in carbonic anhydrase, reduce the catalytic activity of the enzyme and impair enamel mineralization.

Smoking is a major source of exposure to cadmium, especially for people living in areas with low environmental levels of that metal, who have no occupational contact with cadmium [35, 36]. In their study on humans, Han et al. [36] have confirmed a relationship between the blood cadmium levels in smokers and the increased oxidative stress in the oral cavity and periodontium diseases. Active smokers were confirmed by quantifying the cotinine level in urine ≥ 164 ng/mL. The average blood cadmium levels in persons with periodontium diseases were 1.10 *μ*/L in females and 1.24 *μ*g/L in males and were significantly higher by 29% and 32%, respectively, as compared to the average blood cadmium levels in the control population [36].

Thus far, little attention has been paid to the destructive effect of cadmium on salivary glands. The available study outcomes indicate that exposure to that metal may also cause structural and functional changes in those glands, impairing their function and development [7, 34, 37, 38].

Cadmium toxicity derives from its prooxidative properties. Although cadmium is unable to directly generate RFT through Fenton or Haber-Weiss reaction, it does induce stress indirectly through depleting glutathione and other antioxidant in cells, including vitamins, antioxidant enzymes, and bioelement, among those zinc [8, 9, 16, 17, 21], thus impairing the function of numerous tissues and organs, including salivary glands [7, 37, 38]. However, the prooxidative effect of cadmium on salivary glands has not been studied as extensively as its other effects. As a result, it remains insufficiently analyzed and prevents a broader discussion of own research outcomes.

In this study, the long-term exposure to cadmium at both levels induced oxidative stress in the sublingual gland. In the analyzed gland, there was a reduction of the level of nonenzymatic antioxidant (GSH) and of the total antioxidant status (TAS) and an increase of total oxidant status (TOS) and stress indices (LPO, H_2_O_2_, and TOS/TAS). The TAS level was positively correlated to the GSH level (*r* = 0.580) and negatively correlated to TOS (*r* = −0.485), TOS/TAS (*r* = −0.915), LPO (*r* = −0.583), and H_2_O_2_ (*r* = −0.523). On the other hand, TOS and the stress index (TOS/TAS) of the analyzed salivary gland were increased, which in turn conformed to the trend exhibited by LPO (*r* = 0.416; *r* = 0.570) and H_2_O_2_ (*r* = 0.388; *r* = 0.510).

The impact of cadmium on the induction of oxidative stress in the saliva originating from the submandibular gland of rats was studied by Abdollahi et al. [7]. The authors administered i.p. cadmium chloride at 10 mg Cd/kg of body weight and pilocarpine at 8 mg/kg of body weight, as a stimulant of saliva production. Under barbiturate anesthesia, saliva was collected directly from the submandibular gland ducts to microtubes for 30 minutes. In the analyzed material, the TAS level and the complete –SH and LPO groups were assayed. It was determined that exposing rats to cadmium results in a nearly 2-fold reduction of TAS, with simultaneous reduction of the total pool of −SH groups, and 3-fold increase of LPO. In the studies analyzed herein, exposure of rats to 5 mg and 50 mg Cd/dm^3^ resulted in significant decrease of TAS levels by 49% and 44%, respectively, as compared to the control group.

LPO is a lipid peroxidation marker [9, 16]. The majority of researchers quantify the level of malondialdehyde (MDA) as the indicator of oxidative damage to lipid compounds. However, in order to accurately assess this process, LPO levels were assayed, that is, the sum of MDA and 4-hydroxynonenal (4-HNE) [9, 16]. In our own research, the LPO levels were assayed. It increased 2.2-folds at 5 mg Cd/dm^3^ and 3-folds at 50 mg Cd/dm^3^, as compared to the control group, and was higher at a higher exposure level. The increase of LPO level in the sublingual gland at both cadmium exposure levels, as compared to the control group, indicates not only the intensification of lipid peroxidation but also the significant rise of oxidative stress. This is confirmed by the increased level of hydrogen peroxide in the analyzed salivary glands of those rats.

An important indicator of oxidative cellular damage is also the hydrogen peroxide, which is a natural product of cellular metabolism [9]. Due to its strong oxidation properties, that compound is highly reactive and also toxic to the cell. Under normal physiologic conditions, H_2_O_2_ is deactivated by CAT and GPx [9]. Those enzymes prevent excessive accumulation of H_2_O_2_ in cells, thus protecting the organism against the destructive effect of that compound on proteins, lipids, or nucleic acids. It follows from this that the increased H_2_O_2_ levels in the sublingual gland of rats exposed to cadmium, as observed in this study, arise from the inhibition of GPx activity in the analyzed gland, as previously shown in own research [38].

Aside from LPO and H_2_O_2_, another indicator of increased oxidative stress in the cells is TOS and the mathematically calculated TOS/TAS ratio [9]. In the present study, the TOS level and TOS/TAS ratio increased 1.7- and 2.7-folds, respectively, at 5 mg Cd/dm^3^, and 2- and 3-folds, respectively, at higher exposure levels. The obtained results clearly indicate a significantly increased oxidative stress in the sublingual glands of rats exposed to cadmium.

The available study outcomes suggest that antioxidants such as vitamins and polyphenols may protect the organism exposed to cadmium against oxidative stress. Zinc has also been found to have antioxidant properties and has been confirmed to provide efficient protection against many toxic effects of cadmium on the organism, including the damage of the kidneys, liver, and bones [10, 16, 19, 24].

Thus far, the protective role of this bioelement against the consequences of oxidative damage in the sublingual gland of a rat exposed to cadmium has not been studied. This study is a pioneer venture aimed at answering the question whether the increase of zinc supply by 79% and 151%, as compared to standard dietary intake, has protective effect against accumulation of H_2_O_2_ and LPO and low-molecular-weight thiol that is GSH in the analyzed salivary gland.

In the previous own research, we have shown that administering 30 mg Zn/dm^3^ to animals increases the GPx activity, while increasing the supply of that bioelement by 151% (60 mg Zn/dm^3^) increases the activity of both CAT and GPx and reduces the H_2_O_2_ levels, thus confirming zinc's antioxidant effect [38].

This study has shown that administering zinc to animals in both doses during exposure to 5 mg or 50 mg Cd/dm^3^ completely inhibited the cadmium-induced increase of LPO and H_2_O_2_ levels and the reduction of GSH levels in the sublingual gland tissue, allowing us to conclude that zinc supplementation mitigates the oxidative stress in that gland. Furthermore, two-way analysis of variance (ANOVA/MANOVA) has shown that the beneficial effect of zinc supplementation on GSH, LPO, and H_2_O_2_ levels arises from zinc's independent activity and its interaction with cadmium, whereby the independent impact of that bioelement is stronger than the interactive activity between zinc and cadmium.

The role of zinc for oral cavity health has not been extensively studied yet [24, 39–42]. The bioelement is necessary for correct growth of teeth [41], and it reinforces enamel [40, 41] and prevents dental decay [39, 40] and periodontium diseases [39].

Zinc is also used as an antibacterial agent in toothpastes and mouthwashes to control the formation of dental plaque, reduce tartar, and eliminate halitosis [40, 41].

Uckardes et al. [42] assessed the impact of zinc supplementation on the incidence of dental decay in children. 68 children were randomized into 2 groups (study group and placebo group). The children in the study group received 15 mg of zinc daily, five days a week, for 10 weeks. The status of dental plaque was assessed before and after zinc supplementation. The study clearly showed (through reducing the formation of dental plaque in 18 patients) that increased zinc supply prevents dental decay development in children.

Hegde et al. [39] assessed the SOD activity and the Cu and Zn levels in the saliva of patients with and without dental decay. 80 patients were divided according to WHO criteria into healthy patients without decay (DMFT = 0; 20 persons) and patients with active dental decay (DMFT > 10; 60 persons). Saliva was collected always at the same time, that is, at noon, 2 hours after meal. The increase of SOD activity and of copper and zinc levels in the group with active dental decay, achieved by the authors, confirmed that the disease had radical origin.

Saliva is a biological fluid playing an important role for the health of the oral cavity and can be used not only in diagnostics but also in monitoring the progress of various diseases, including cancer and periodontitis [33, 43–45].

Wei et al. [45] assessed the TOS and MDA levels and SOD activity in the serum, saliva, and gingival crevicular fluid before and 16 weeks after conventional therapy (maintaining appropriate oral cavity hygiene and cleaning gum surface) of chronic periodontitis. The study involved 83 patients, of which 48 had manifest symptoms. Lipid peroxidation assessed as MDA level was higher before therapy only in the gingival crevicular fluid and was reduced by treatment. The SOD activity and total oxidation status of the analyzed biological fluids were higher before therapy and were reduced by treatment. The outcomes of that study indicate that oxidative stress might play an important role in chronic periodontitis and appropriate therapy may help control the process by modifying SOD activity and MDA and TOS levels.

In other studies, Kurku et al. [44] assessed the oxidative stress indices in smokers' serum and saliva. The MDA, TOS, and TOS/TAS ratio were higher in the smokers' serum, while the total level of the –SH groups decreased. In the smokers' saliva, a decrease of activity of the GPx and total –SH groups was observed, coupled with an increase of MDA levels. The authors suggest that disrupting the oxidation-reduction balance of saliva and serum arises from smoking and has destructive effect on tissues and organs, including the oral cavity.

The key outcome of our own research is the confirmation that administering zinc to animals at both concentration levels during exposure to cadmium significantly increases the TAS and reduces the TOS, as well as the TOS/TAS stress index in the sublingual glands of rats, which suggests a reduction of oxidative stress in that gland. Two-way analysis of variance (ANOVA/MANOVA) has shown that the beneficial effect of zinc supplementation on TAS and TOS levels and TOS/TAS ratio arises from zinc's independent activity and its interaction with cadmium, whereby the independent impact of that bioelement is stronger than the interactive activity between zinc and cadmium.

## 5. Conclusions

In light of the available literature data on the protective effect of zinc, it should be noted that this study is the first attempt to analyze the impact of that bioelement on the sublingual gland tissue during exposure to cadmium. Summing up the outcomes of our own research, it should be concluded that a confirmed increase, in the applied experimental model for both the moderate environmental exposure of humans to cadmium and the occupational exposure, of the zinc supply by 79% and 151% reduces the oxidative stress in sublingual glands of rats and clearly shows a protective effect of that bioelement on the tissue of the analyzed gland.

The outcome of this study contributes new, important data confirming the protective effect of increased zinc intake on the sublingual gland tissue in chronic exposure to cadmium.

## Figures and Tables

**Table 1:** The impact of zinc on GSH, LPO, and H_2_O_2_ levels in the sublingual gland of rats exposed to cadmium.

	Nonenzymatic antioxidant GSH (nmol/mg of protein)	Oxidative stress index	
		LPO (nmol/mg of protein)	H_2_O_2_ (nmol/mg of protein)
Control	1.881 ± 0.800	0.122 ± 0.011	21.320 ± 2.343
30 mg Zn/dm^3^	1.926 ± 0.181	0.095 ± 0.008	19.110 ± 0.617
60 mg Zn/dm^3^	1.838 ± 0.570	0.093 ± 0.015	15.740 ± 1.336^a^^∗^
5 mg Cd/dm^3^	1.433 ± 0.086^a‡b‡c^^∗^	0.263 ± 0.034^a†b‡c†^	26.930 ± 1.000^a^^∗^^b‡^
5 mg Cd/dm^3^ + 30 mg Zn/dm^3^	1.933 ± 0.136^d†^	0.113 ± 0.020^d†^	17.480 ± 0.720^d‡^
5 mg Cd/dm^3^ + 60 mg Zn/dm^3^	2.334 ± 0.144^a†b^^∗^^c†d‡e†^	0.104 ± 0.012^d†^	18.020 ± 1.248^d‡^
50 mg Cd/dm^3^	1.340 ± 0.046^a†b‡c†e‡f‡^	0.376 ± 0.066^a‡b‡c‡d^^∗^^e‡f‡^	41.800 ± 2.877^a‡b‡d‡f‡^
50 mg Cd/dm^3^ + 30 mg Zn/dm^3^	1.715 ± 0.067^f‡g^^∗^	0.233 ± 0.045^a^^∗^^b†c†f^^∗^^g†^	21.860 ± 1.480^d^^∗^^g‡^
50 mg Cd/dm^3^ + 60 mg Zn/dm^3^	1.739 ± 0.052^f‡g^^∗^	0.167 ± 0.023^d^^∗^^e^^∗^^g‡^	17.540 ± 1.963^d‡g‡^

The values are arithmetic means ± SEM. ^∗^*p* < 0.05; ^†^*p* < 0.01; ^‡^*p* < 0.001 as compared to ^a^control, ^b^30 mg Zn/dm^3^, ^c^60 mg Zn/dm^3^, ^d^5 mg Cd/dm^3^, ^e^5 mg Cd/dm^3^ + 30 mg Zn/dm^3^, ^f^5 mg Cd/dm^3^ + 60 mg Zn/dm^3^, ^g^50 mg Cd/dm^3^, and ^h^50 mg Cd/dm^3^ + 30 mg Zn/dm^3^.

**Table 2:** The impact of zinc on TOS and TAS levels, and the TOS/TAS ratio in the sublingual gland of rats exposed to cadmium.

	TOS (nmol/mg of protein)	TAS (nmol/mg of protein)	TOS/TAS
Control	21.846 ± 3.751	3.466 ± 0.468	7.709 ± 1.971
30 mg Zn/dm^3^	16.393 ± 1.157	7.843 ± 0.590^a‡^	2.181 ± 0.236^a†^
60 mg Zn/dm^3^	17.951 ± 0.985	6.138 ± 0.839^a‡b^^∗^	3.272 ± 0.435^a^^∗^
5 mg Cd/dm^3^	36.284 ± 3.438^a‡b‡c‡^	1.770 ± 0.080^a^^∗^^b‡c‡^	20.565 ± 1.796^a‡b‡c‡^
5 mg Cd/dm^3^ + 30 mg Zn/dm^3^	19.700 ± 1.557^d‡^	7.879 ± 0.379^a‡c^^∗^^d‡^	2.515 ± 0.519^a†d‡^
5 mg Cd/dm^3^ + 60 mg Zn/dm^3^	20.974 ± 1.626^d‡^	12.009 ± 0.559^a‡b‡c‡d‡e‡^	1.777 ± 0.157^a†d‡^
50 mg Cd/dm^3^	44.153 ± 3.943^a‡b‡c‡d^^∗^^e‡f‡^	1.930 ± 0.213^a^^∗^^b‡c‡e‡f‡^	24.208 ± 2.543^a‡b‡c‡d^^∗^^e‡f‡^
50 mg Cd/dm^3^ + 30 mg Zn/dm^3^	21.205 ± 2.054^d‡g‡^	5.382 ± 0.627^a^^∗^^b†d‡e†f‡g‡^	4.093 ± 0.411^d‡g‡^
50 mg Cd/dm^3^ + 60 mg Zn/dm^3^	26.684 ± 2.313^b^^∗^^c^^∗^^d†g‡^	5.104 ± 0.454^a^^∗^^b‡d‡e‡f‡g‡^	5.433 ± 0.533^d‡g‡^

The values are arithmetic means ± SEM. ^∗^*p* < 0.05; ^†^*p* < 0.01; ^‡^*p* < 0.001 as compared to ^a^control, ^b^30 mg Zn/dm^3^, ^c^60 mg Zn/dm^3^, ^d^5 mg Cd/dm^3^, ^e^5 mg Cd/dm^3^ + 30 mg Zn/dm^3^, ^f^5 mg Cd/dm^3^ + 60 mg Zn/dm^3^, ^g^50 mg Cd/dm^3^, and ^h^50 mg Cd/dm^3^ + 30 mg Zn/dm^3^.

**Table 3:** Independent and interactive impact of cadmium and zinc on the levels of selected oxidative stress indices in the rat sublingual gland.

ANOVA/MANOVA	GSH	LPO	H_2_O_2_	TOS	TAS	TOS/TAS
Independent impact of Cd	6.389^∗^	24.84^‡^	20.38^‡^	35.05^‡^	0.635	62.04^‡^
Independent impact of Zn	9.526^†^	14.09^‡^	37.82^‡^	33.31^‡^	53.18^‡^	149.5^‡^
Interaction effect of Cd and Zn	9.461^†^	7.091^†^	13.67^‡^	11.56^†^	3.049	50.87^‡^

The values reflect the *F* coefficient, where ^∗^*p* < 0.05; ^†^*p* < 0.01; ^‡^*p* < 0.001.

**Table 4:** Analysis of Spearman's rank correlation between the assessed parameters in the sublingual gland tissue.

	TAS	TOS	TOS/TAS	GSH	H_2_O_2_
TAS	—				
TOS	−0.485^‡^	—			
TOS/TAS	−0.915^‡^	0.756^‡^	—		
GSH	0.580^‡^	−0.431^‡^	−0.588^‡^	—	
H_2_O_2_	−0.523^‡^	0.388^‡^	0.510^‡^	−0379^†^	—
LPO	−0.583^‡^	0.416^‡^	0.570^‡^	−0.503^‡^	0.501^‡^

The values reflect the rank correlation coefficient *r*, where ^†^*p* < 0.01; ^‡^*p* < 0.001.

## Data Availability

The data of the materials and methods and conclusions to support the findings of this study are included within the article. If any other data may be needed, please contact the corresponding author upon request.
